# Neurotrophin-4 promotes *in vitro* development and maturation of human secondary follicles yielding metaphase II oocytes and successful blastocyst formation

**DOI:** 10.1093/hropen/hoae005

**Published:** 2024-01-30

**Authors:** Yingchun Guo, Lei Jia, Haitao Zeng, Peng Sun, Wenlong Su, Tingting Li, Xiaoyan Liang, Cong Fang

**Affiliations:** Reproductive Medicine Research Center, The Sixth Affiliated Hospital, Sun Yat-Sen University, Guangdong, Guangzhou, China; GuangDong Engineering Technology Research Center of Fertility Preservation, Guangdong, Guangzhou, China; Reproductive Medicine Research Center, The Sixth Affiliated Hospital, Sun Yat-Sen University, Guangdong, Guangzhou, China; GuangDong Engineering Technology Research Center of Fertility Preservation, Guangdong, Guangzhou, China; Reproductive Medicine Research Center, The Sixth Affiliated Hospital, Sun Yat-Sen University, Guangdong, Guangzhou, China; GuangDong Engineering Technology Research Center of Fertility Preservation, Guangdong, Guangzhou, China; Reproductive Medicine Research Center, The Sixth Affiliated Hospital, Sun Yat-Sen University, Guangdong, Guangzhou, China; GuangDong Engineering Technology Research Center of Fertility Preservation, Guangdong, Guangzhou, China; Reproductive Medicine Research Center, The Sixth Affiliated Hospital, Sun Yat-Sen University, Guangdong, Guangzhou, China; GuangDong Engineering Technology Research Center of Fertility Preservation, Guangdong, Guangzhou, China; Reproductive Medicine Research Center, The Sixth Affiliated Hospital, Sun Yat-Sen University, Guangdong, Guangzhou, China; GuangDong Engineering Technology Research Center of Fertility Preservation, Guangdong, Guangzhou, China; Reproductive Medicine Research Center, The Sixth Affiliated Hospital, Sun Yat-Sen University, Guangdong, Guangzhou, China; GuangDong Engineering Technology Research Center of Fertility Preservation, Guangdong, Guangzhou, China; Reproductive Medicine Research Center, The Sixth Affiliated Hospital, Sun Yat-Sen University, Guangdong, Guangzhou, China; GuangDong Engineering Technology Research Center of Fertility Preservation, Guangdong, Guangzhou, China

**Keywords:** human follicle, *in vitro* culture, neurotrophin-4, blastocyst, fertility preservation, prepubertal patient

## Abstract

**STUDY QUESTION:**

Does a matrix-free culture system supplemented with neurotrophic factor 4 (NT4) improve human *in vitro* follicular development and meiotic maturation, ultimately resulting in fertilizable oocytes?

**SUMMARY ANSWER:**

NT4 supplementation of *in vitro* culture significantly enhances the growth, steroid hormone production, and maturity potential of human secondary follicles derived from fresh ovarian medulla (from post- and pre-pubertal patients), thereby yielding fertilizable oocytes.

**WHAT IS KNOWN ALREADY:**

Reconstituting folliculogenesis *in vitro* is of paramount importance in the realms of fertility preservation, reproductive biology research, and reproductive toxicity assessments. However, the efficiency of *in vitro* culture systems remains suboptimal, as the attainment of fertilizable oocytes from *in vitro* growth (IVG) of human follicles remains unachieved, with the data being particularly scant regarding follicles from prepubertal girls. We have previously found that mouse oocytes from secondary follicles derived from IVG are deficient in neuroendocrine regulation. NT4 and its corresponding receptor have been identified in human follicles. Significantly, the addition of NT4 during the IVG process markedly enhances both follicle growth and oocyte maturation rates in mice.

**STUDY DESIGN, SIZE, DURATION:**

Fresh medulla tissue obtained during tissue preparation for ovarian tissue cryopreservation (OTC) were collected from 10 patients aged from 6 to 21 years old, all of whom had undergone unilateral oophorectomy as a means of fertility preservation. Isolated secondary follicles were individually cultured *in vitro* with or without NT4 in a matrix-free system.

**PARTICIPANTS/MATERIALS, SETTING, METHODS:**

Secondary follicles, extracted via enzymatic digestion and mechanical disruption from each patient, were randomly allocated to either a control group or an NT4-supplemented group (100 ng/ml), followed by individual culture on an ultra-low attachment plate. Follicle growth and viability were assessed by microscopy. Levels of anti-Müllerian hormone (AMH), estradiol, and progesterone in the medium were quantified. An oocyte-specific marker was identified using confocal fluorescence microscopy following DEAD box polypeptide 4 (DDX4) staining. The competence of individual oocytes for maturation and fertilization were assessed after IVM and ICSI with donated sperm samples.

**MAIN RESULTS AND THE ROLE OF CHANCE:**

Overall, isolated follicles from both groups survived up to 6 weeks with increasing diameters over the duration (*P* < 0.05), reaching terminal diameters of almost 1 mm with confirmed steroidogenesis and expression of oocyte marker (DDX4), and producing morphologically normal MII oocytes. When compared with the control group, the NT4 group had a similar initial follicular diameter (206 ± 61.3 vs 184 ± 93.4 μm) but exhibited a significant increase in follicular diameter from the ninth day of culture onwards (*P* < 0.05). From Week 3, estradiol and progesterone production were significantly increased in the NT4 group, while no significant difference was observed in AMH production between groups. The proportion of ‘fast-growth’ follicles in the NT4 group was significantly higher than that in the control group (13/23 vs 6/24, *P* < 0.05). An increased efficiency of MII oocyte maturation per live follicle in the NT4 group was also observed (control group vs NT4 group, 4/24 vs 10/23, *P* < 0.05). It is noteworthy that an MII oocyte obtained from the control group exhibited abnormal fertilization after ICSI. In contrast, an MII oocyte acquired from the NT4 group progressed to the blastocyst stage and showed potential for transfer.

**LARGE SCALE DATA:**

N/A.

**LIMITATIONS, REASONS FOR CAUTION:**

The cohort examined in this study was all patients diagnosed with beta-thalassemia major. Whether this culture system is effective for patients with other diseases remains unknown. Since the chosen dose of NT4 was established based on dose finding in mice, the optimal dose for use in a human IVG system needs further confirmation. The oocytes and embryos procured from this study have not been quantified for ploidy status or epigenetic signatures.

**WIDER IMPLICATIONS OF THE FINDINGS:**

Fresh medulla tissue obtained during tissue preparation for OTC may serve as a precious source of fertilizable oocytes for female fertility preservation, even for pre-pubertal girls, without the threat of tumour reintroduction. After further characterization and optimization of the system, this culture system holds the potential to provide a powerful future research tool, for the comprehensive exploration of human follicular development mechanisms and for conducting reproductive toxicity evaluations.

**STUDY FUNDING/COMPETING INTEREST(S):**

This work was supported by the National Key R&D Program of China (grant number 2022YFC2703000) and National Natural Science Foundation of China (grant numbers 82271651 and 81871214). The medium used in human follicle *in vitro* culture in this study has been applied for a national invention patent in China (No. 202211330660.7). The inventors of the patent, in order, are: Y.G., C.F., and X.L.

WHAT DOES THIS MEAN FOR PATIENTS? As more people survive cancer, a growing number of women are finding that their ability to have children in the future is affected either by the cancer itself or by the treatments they receive. For these women, it is becoming very important to find ways to preserve their ability to have children. One exciting new method being explored is growing their follicles in a lab in a process called *in vitro* follicle growth or IVG. This method could help save more oocytes for future use and also avoids the risk of reintroducing the cancer to the patient because they would not need to be transplanted.

However, this method is not yet perfect. We are still trying to improve how it works to make sure it can produce oocytes that can lead to a pregnancy. In our research, we used tissue that is usually thrown away when preparing ovarian tissue for freezing in the process of fertility-preservation. We tried to grow follicles from this tissue in the lab, adding a special substance (neurotrophic factor 4 or NT4) to see if it helps.

Our findings are promising. Adding NT4 seems to help these follicles grow better and produce the necessary hormones, and for the oocytes to reach a stage where they could potentially be fertilized. This is true for tissue from both young girls and adult women. This approach could be a valuable way to help women, including young girls, preserve their ability to have children in the future, without the risk of reintroducing their cancer. Our work is not just about helping individuals; it also opens up new ways to study how human follicles and oocytes develop and to test how different treatments might affect fertility.

## Introduction

Reconstituting folliculogenesis *in vitro* is important for fertility preservation, reproductive biology research, and assessments of reproductive toxicity. Since the long-term survival rate of females receiving gonadotoxic treatment is greater than ever before, fertility preservation (FP) has gained prominence (Anderson *et al.*, 2020; Reynolds and McKenzie, 2023). As the only FP method available for prepubertal girls or females constrained by time or contraindications against oocyte cryopreservation (Gellert *et al.*, 2018; Khattak *et al.*, 2022), ovarian tissue cryopreservation (OTC) was no longer deemed experimental since 2019 (Practice Committee of the American Society for Reproductive Medicine, 2019). Although early-growing follicles can resume *in vivo* development after ovarian tissue autologous transplantation, the potential reintroduction of malignant cells in certain cancers and the compromised ovarian reserve due to cryopreservation and pre-vascularization phases pose significant challenges. *In vitro* follicle growth (IVG) emerges as an alternative avenue to procure mature oocytes from ovarian tissue without necessitating hormone stimulation, benefiting both young women and girls. Crucially, IVG circumvents the risk of cancer cell reintroduction associated with auto-transplantation. Healthy offspring have been achieved after the IVG of mouse secondary follicles derived from vitrified (Wang *et al.*, 2011) or fresh (Xu *et al.*, 2006) ovarian tissue, and live offspring have been produced from mouse primordial follicles with a multistep protocol (Eppig and O’Brien, 1996; O’Brien *et al.*, 2003). Nevertheless, IVG for human follicles remains a great challenge due to the larger follicle size and extended culture durations. To date, only three human follicle culture systems have yielded MII oocytes (Xiao *et al.*, 2015; McLaughlin *et al.*, 2018; Xu *et al.*, 2021), with maturation rates ranging from 10% (9/87) to 21% (3/14). Optimizing the culture system to enhance the yield of mature, fertilizable oocytes remains a critical objective.

The key difference between IVG and the *in vivo* environment is the breakthrough point. Follicle growth *in vivo* is a dynamic but protracted process, regulated by a combination of endocrine, paracrine, and autocrine interactions (Demeestere *et al.*, 2005; Bernabe *et al.*, 2020). Previously (Guo *et al.*, 2021), we delineated the transcriptomic differences between mouse oocytes from *in vivo* developed antral follicles and those developed *in vitro* from the secondary stage. Our findings highlighted a notable deficit in neuroendocrine regulation for oocytes from IVG follicles. Accumulating evidence has suggested neurotrophins play important roles in follicle assembly, follicular development, and oocyte maturation (Streiter *et al.*, 2016; Chang *et al.*, 2019). Neurotrophic factor 4 (NT4), a member of the neurotrophin family, has been identified to be distinctly stage-specific and preferentially expressed in the oocytes of human preantral and antral follicles (Zhang *et al.*, 2018). However, whether NT4 could improve human follicular development and allow the achievement of fertilizable oocytes *in vitro* remains unknown. Furthermore, although pre-pubertal patients account for a large proportion of people with indications for fertility preservation, very little is known about their ovaries and the IVG effectiveness for them.

In this study, the matrix-free *in vitro* culture system of human secondary follicles was constructed with fresh medulla tissue obtained during tissue preparation for OTC for post- and pre-pubertal patients. NT4 was supplemented to test its effect on optimization. Follicle survival and growth, production of steroid hormones and paracrine factors, oocyte marker expression, as well as oocyte maturity and fertility potential were assessed.

## Materials and methods

### Ethical approval and reagents

In clinical settings, only cortical tissue is cryopreserved for FP (Oktay *et al.*, 2018). Fresh medulla tissue, obtained during tissue preparation for OTC, was collected for this study. The experimental protocol and the use of human donated sperm received approval from the ethics committee of the Sixth Affiliated Hospital of Sun Yat-Sen University (2021ZSLYEC-521, 2017ZSLYEC-015S). All women or parents/guardians of girls under the age of 18 years had signed the informed consent and were informed about the possible generation of embryos for research only. All the reagents were purchased from Sigma (St Louis, MO, USA) unless otherwise stated.

### Ovarian tissue collection

Unilateral ovaries were taken from 10 patients for fertility preservation, immersed in Quinn’s Advantage™ Medium with HEPES (SAGE, Trumbull, CT, USA) supplemented with 10% serum protein substitute (SPS, SAGE), kept at 4 °C and transferred to the laboratory within 1 h.

### Follicle isolation

During tissue preparation for OTC, fresh medulla tissue was trimmed off and collected, and chopped with ophthalmic scissors swiftly until adjusted to 0.5 × 0.5 × 0.5 mm^3^. Approximately 1.5 g tissues were digested in 10 ml medium consisting of Leibovitz’s L-15 (L-15, Invitrogen, Carlsbad, CA, USA) containing 0.2 mg/ml DNase I (Worthington Biochemical Corporation, Lakewood, NJ, USA), 0.2 mg/ml Collagenase IV (BioFroxx, Einhausen, Germany), and 0.04 mg/ml Liberase TM (Roche, Mannheim, Germany), as previously described with modifications (Yin *et al.*, 2016). After incubation at 37 °C in the incubator (5% CO_2_ in air, ASTEC, Fukuoka, Japan) for 60 min, the digestion was terminated by the addition of an equal volume of L-15 supplemented with 10% SPS at 37 °C. After being washed twice in dissection medium (DM), which comprises L-15 medium supplemented with 10% SPS, the tissue samples were dissected with insulin syringes in pre-warmed DM on a heated stage maintained at 37 °C, under a dissecting microscope. Secondary follicles with a centrally located oocyte, intact basement membrane and no signs of somatic cell degeneration, were picked for subsequent culture.

### Individual follicle culture

Basic culture media consisted of αMEM (Gibco, Carlsbad, CA, USA) containing 6% SPS, 1 μl/ml insulin–transferrin–selenium (ITS), 1 mg/ml fetuin, and 10 mIU/ml rhFSH (Gonal-f, MERCK, Darmstadt, Germany). Follicles from each patient were randomly assigned to two study groups and transferred to the corresponding culture media: the control group with basic culture media, and the NT4 group supplemented with 100 ng/ml human NT4 (PeproTech, Rocky Hill, NJ, USA) (Application number of national invention patent of China: 202211330660.7). The chosen concentration of 100 ng/ml was based on a previous study in mice, which was shown to be superior in improving follicle diameter and oocyte maturation in secondary follicle IVG (Guo *et al.*, 2021). Experiments for the two groups were conducted in parallel. Each follicle was individually incubated in the wells of round-bottom ultra-low attachment microplates (Catalog #7007, Corning, New York, NY, USA) in 200 μl of pre-equilibrated and pre-warmed culture medium at 37 °C and 5% CO_2_ for 4–6 weeks. Medium refreshment was performed every other day by replacing half of the corresponding culture media. The spent medium was collected and stored at −80°C for subsequent assays.

### Follicle survival and growth measurements

The morphology of follicles was photographed with a Nikon TS100 light microscope (Nikon Instruments, Inc., Tokyo, Japan) equipped with a LYKOS imaging system, and diameters were calculated by averaging the two widest perpendicular measurements taken from follicle peripheries with Image J software every other day. Survival data were reported on Week 4 of culture. Follicles were considered degenerated if the oocyte shrank, a denuded oocyte hatched from the follicle, or follicle diameter growth became stagnant for one week. The initiation of antrum formation within the follicle was determined by a visible translucent area around the oocyte.

### Estradiol-17 β, AMH, and progesterone measurement

Media samples were pooled by week for each patient. Measurements were performed with an automatic electrochemiluminescence immunoassay instrument (Cobas e601, Roche, Basel, Switzerland) according to the manufacturer's instructions. The detection ranges for estradiol-17 β, anti-Müllerian hormone (AMH), and progesterone were 5–3000 pg/ml, 0.01–23 ng/ml, and 0.05–60 ng/ml, respectively. Intra- and inter-assay coefficients of variation were 6.7% and 10.6% for estradiol-17 β, 1.7% and 3.5% for AMH, and 11.9% and 22.5% for progesterone. Three samples were analysed per condition.

### Immunofluorescence of oocytes

As a biological control, DEAD box polypeptide 4 (Ddx4) (Fujiwara *et al.*, 1994; Castrillon *et al.*, 2000), an evolutionarily conserved germ cell-specific RNA helicase, was detected in oocytes from the IVG system. Three oocytes were assessed in both groups. Germinal vesicle (GV) oocytes that failed to mature after oocyte IVM were fixed in 4% paraformaldehyde (Servicebio, Wuhan, China) for 30 min, and then transferred to drops of 0.5% TRITON X-100 (ZSGB-BIO, Beijing, China) for 30 min. Oocytes were blocked in 3% BSA diluted in phosphate-buffered saline (PBS; pH 7.4, Gibco). One hour later, oocytes were incubated with anti-DDX4 antibody (1:200; ABCAM; Cambridge, UK) overnight at 4 °C followed by incubation with Alexa Fluor 647 goat anti-mouse IgG (1:500; Thermo Fisher, Carlsbad, CA, USA) for 1 h. Cell nuclei were stained with DAPI dihydrochloride (Invitrogen). Confocal images were acquired using a Leica Corp. TCS SP8 confocal system (Leica Corp. Microsystems, Heidelberg, Germany).

### Oocyte IVM

When no sign of follicle degeneration was detected at Week 6 of culture, or the follicle reached a diameter of 800 μm, follicles were cultured individually in 100 μl pre-equilibrated pre-IVM medium for 4 h (Zeng *et al.*, 2013; Gilchrist *et al.*, 2016), followed by rinsing and culture in pre-equilibrated IVM medium in the wells of round bottom ultra-low attachment microplates (Catalog #7007, Corning) at 37 °C (5% CO_2_ and 20% O_2_). The pre-IVM medium contained M199 medium supplemented with 50 µM Forskolin, 50 µM IBMX, 100 mIU/ml r-FSH (Gonal-f, MERCK), 5 mg/ml HSA, 0.6 mM L-cysteine, 0.91 mM sodium pyruvate, 50 μg/ml penicillin, and 75 μg/ml streptomycin. The IVM medium was composed of M199 medium supplemented with 100 mIU/ml r-FSH, 10 ng/ml EGF, 5 μg/ml insulin, 5 mg/ml HSA, 0.6 mM L-cysteine, 0.91 mM sodium pyruvate, 50 μg/ml penicillin, and 75 μg/ml streptomycin. After 24–48 h, COCs were gently dissected out from follicles using needles and cumulus cells were stripped off gently in HEPES containing 80 IU/ml of hyaluronidase (Vitrolife, Gothenburg, Sweden). The appearance of the first polar body served as a marker for maturation. GV oocytes that failed to mature following IVM were either utilized for immunofluorescence testing or discarded.

### ICSI and embryo culture

Mature oocytes fertilized by ICSI with donated sperm, were cultured in G-SERIES culture medium (Vitrolife). Normal fertilization was verified by the presence of two pronuclei according to the Istanbul consensus (Alpha Scientists in Reproductive Medicine and ESHRE Special Interest Group of Embryology, 2011). Time-lapse (Embryoscope, Unisense FertiliTech A/S, Midtjylland, Denmark) was administrated to observe the cellular behaviour, imaging embryos every 15 min. At least two experienced embryologists assessed the morphokinetic parameters according to the definitions from the consensus paper (Ciray *et al.*, 2014), and graded embryos according to criteria modified from the grading system described by Racowsky (blastomere) (Racowsky *et al.*, 2003), Sakkas and Gardner (blastocyte) (Sakkas and Gardner, 2005).

### Statistical analyses

Analyses were performed with the SPSS 23.0 program (SPSS Inc., Chicago, IL, USA). The diameter and hormone production data are presented as means±SDs and analysed with Student’s t-test or Mann-Whitney U test for comparisons between two groups. Rates of survival and IVM are presented as n/n and analysed with the chi-square test, Yates’ correction, or Fisher’s exact probabilities. *P *<* *0.05 was considered statistically significant.

## Results

### Characteristics of patients

Fresh medulla tissue samples were collected from ten beta-thalassemia major patients aged 6–21 years. Among these patients, 80% were premenarcheal and 20% were postmenarcheal (Table 1). None of the patients had undergone radiation and/or chemotherapy prior to ovarian tissue removal.

**Table 1.** Characteristics of patients.

Patient	Age	Diagnosis	Menarchal status	All follicles (n)	Group	Follicles at the start of culture (n)	Follicles survived (n)	Mature oocytes (n)
No. 1	11	Beta-thalassemia major	Premenarche	16	IVG_C	8	3	1
					IVG_NT	8	2	1
No. 2	10	Beta-thalassemia major	Premenarche	13	IVG_C	6	1	0
					IVG_NT	7	2	1
No. 3	21	Beta-thalassemia major	Postmenarche	12	IVG_C	6	2	1
					IVG_NT	6	2	1
No. 4	13	Beta-thalassemia major	Premenarche	11	IVG_C	5	2	0
					IVG_NT	6	2	1
No. 5	19	Beta-thalassemia major	Postmenarche	19	IVG_C	9	3	1
					IVG_NT	10	4	2
No. 6	11	Beta-thalassemia major	Premenarche	18	IVG_C	9	3	1
					IVG_NT	9	3	1
No. 7	6	Beta-thalassemia major	Premenarche	12	IVG_C	6	2	0
					IVG_NT	6	2	1
No. 8	8	Beta-thalassemia major	Premenarche	11	IVG_C	6	2	0
					IVG_NT	5	1	0
No. 9	9	Beta-thalassemia major	Premenarche	13	IVG_C	7	3	0
					IVG_NT	6	2	1
No. 10	12	Beta-thalassemia major	Premenarche	16	IVG_C	8	3	0
					IVG_NT	8	3	1

IVG_C group, follicles cultured *in vitro* without neurotrophic factor 4; IVG_NT group, follicles cultured *in vitro* with neurotrophic factor 4.

### Follicle survival, growth, and the effect of NT4 supplementation

**Figure 1.**
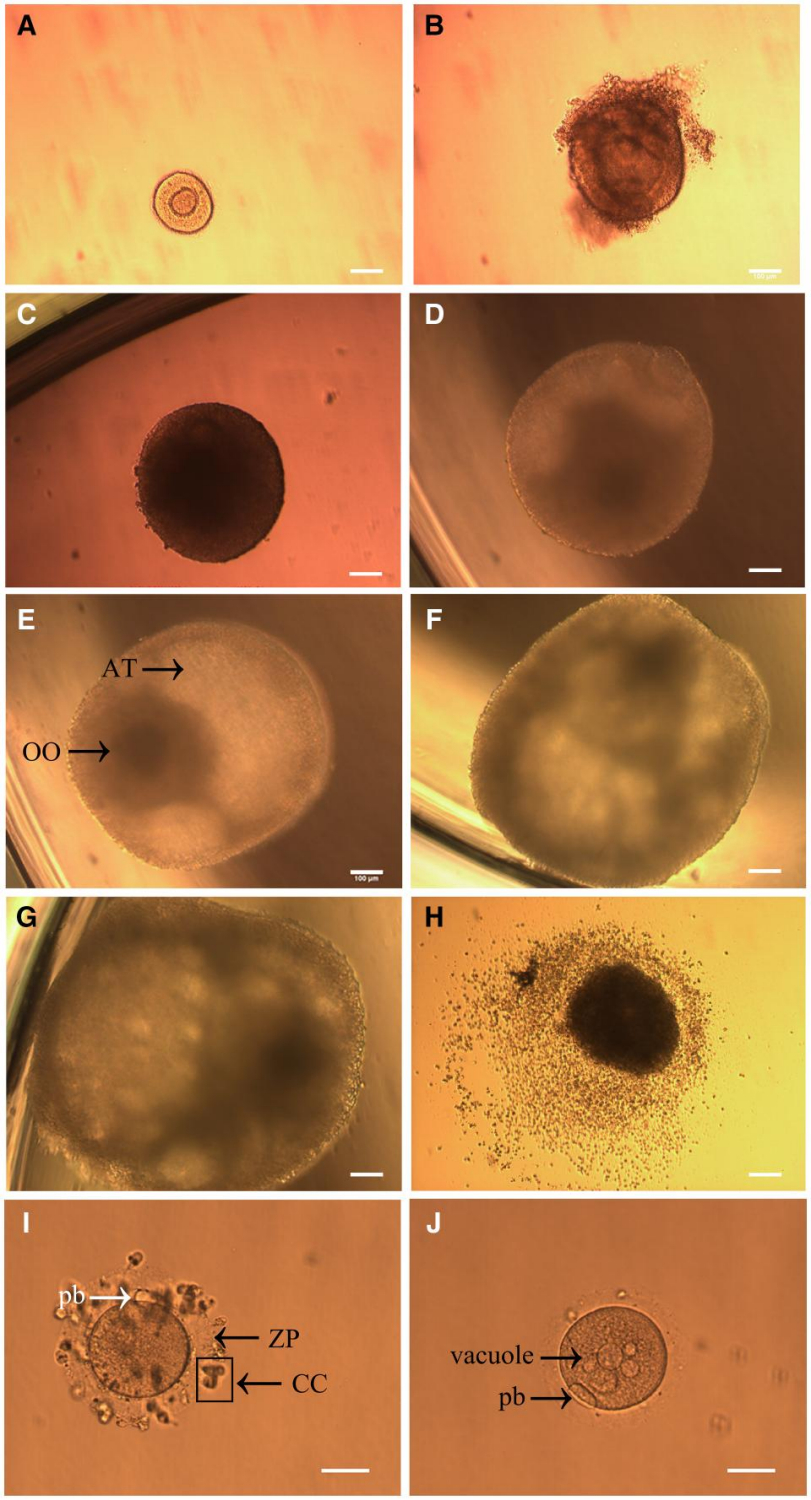
**Representative images of human secondary follicles cultured *in vitro* and captured by optical microscopy.** Secondary follicle isolated from ovarian medulla tissue (**A**). The growth of a secondary follicle *in vitro* on Week 1 (**B**), Week 2 (**C**), Week 3 (**D**), Week 4 (**E**), Week 5 (**F**), and Week 6 (**G**). Bar = 100 μm. Morphology of cumulus–oocyte complexes (COCs) after IVM culture (**H**). Morphology of human metaphase II oocytes (**I, J**) derived from *in vitro*-cultured antral follicles. Bar = 50 μm. Cultured antral follicles exhibited a 3-dimensional morphology similar to that of *in vivo* follicles, including an antrum (AT) and an oocyte (OO) (**E**). The first polar body (pb), cumulus cells (CC), and zona pellucida (ZP) were observed with normal sizes and positions in the metaphase II oocytes harvested from *in vitro*-cultured antral follicles and matured *in vitro* (**I, J**). One of the metaphase II oocytes derived from a follicle cultured *in vitro* in the control medium contained vacuoles in the cytoplasm (**J**).

**Figure 2.**
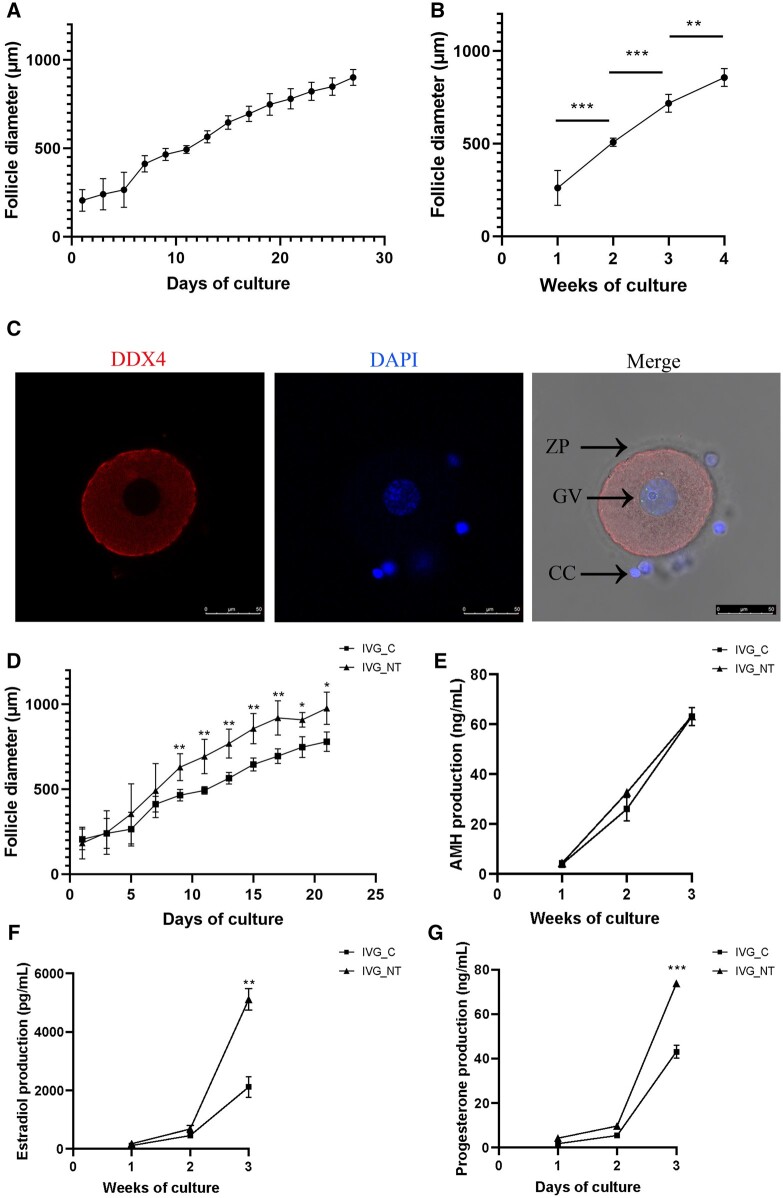
**Diameter, hormone production, and expression of an oocyte marker gene (DDX4) in human follicles cultured *in vitro*, and the effect of neurotrophic factor 4 (NT4) supplementation.** (**A**) A linear growth in diameter. (**B**) The average follicle diameter increased each week from the previous week in the control medium (*P* < 0.05). (**C**) As shown by immunofluorescence staining, oocytes harvested from *in vitro*-cultured follicles exhibited a germinal vesicle nucleus (GV) and zona pellucida (ZP), surrounded by cumulus cells (CC). DDX4 staining (red) delineates its cytoplasmic localization in the oocytes. DAPI staining (blue) depicts chromatin in the nucleus of oocyte and cumulus cells. (**D**) Dynamics of follicle diameter in culture *in vitro* with (IVG_NT) or without (IVG_C) supplementation of NT4. Hormone profiles of AMH (**E**), estradiol (**F**), and progesterone (**G**) in the IVG_C group and IVG_NT group (100 ng/ml) during *in vitro* culture. Data are presented as the mean±SD. *P < 0.05, **P < 0.01, ***, *P* < 0.001. Bar = 50 μm. AMH, anti-Müllerian hormone.

A total of 141 secondary follicles were isolated, with follicles from each patient being randomly assigned to the two study groups (Table 1). An average of 14.4 follicles were obtained per patient (range: 11–19). Across both study groups, the isolated secondary follicles survived for up to 6 weeks, displaying a steady, linear increase in diameter and forming an antrum between Weeks 2 and 3 (Figs 1A–D and 2A, B, and D). The cultured antral follicles exhibited a 3-dimensional spherical morphology similar to *in vivo* follicles, characterized by features such as an antral cavity, COC, intact granulosa layers, and a basement membrane (Fig. 1E–G). For follicles that survived, all weekly increases in diameter were statistically significant (*P* < 0.05, Fig. 2B). Among the secondary follicles which had survived until Week 4, two distinct growth patterns were identified: ‘fast-growth’ follicles. which expanded their diameters by a minimum of 3-fold at the third week of IVG, and ‘slow-growth’ follicles, whose diameter increase was less than 3-fold at the third week of IVG.

No significant difference was found in the average diameter at the onset of culture between the groups (control group vs NT4 group, 206 ± 61.3 vs 184 ± 93.4 μm, *P* > 0.05). From Day 9, diameters in the NT4 group were notably higher than those in the control group (631 ± 79.4 vs 466 ± 34.1 μm, *P* < 0.05, Fig. 2D). The proportion of ‘fast-growth’ follicles in the NT4 group was significantly higher than that in the control group (13/23 vs 6/24, *P* < 0.05, Table 2). We observed no difference in the survival rate between the groups at Week 4 of follicle IVG (Table 2). At the end of follicle culture (before IVM), surviving follicles reached terminal diameters of 965 ± 32.0 and 969 ± 73.1 μm in the control group and NT4 group, respectively (*P*>0.05).

**Table 2.** *In vitro* growth and IVM of human secondary follicles.

	IVG_NT	IVG_C
Number of follicles cultured	71	70
Initial diameters (μm)	184 ± 93.4	206 ± 61.3
Rates of follicles attached with stromal cells at the beginning of culture; % (n/n)	91.5 (65/71)	88.6 (62/70)
Diameters on Day 9 (μm)*	631 ± 79.4	466 ± 34.1
Proportion of ‘fast-growth’ follicles*; % (n/n)	56.5 (13/23)	25 (6/24)
Survival rate; % (n/n)	32.4 (23/71)	34.3 (24/70)
Antrum formation rates for those surviving until Week 4; % (n/n)	100 (23/23)	100 (24/24)
Terminal diameters (μm, before IVM)	969 ± 73.1	965 ± 32.0
Maturation rate per live follicle*; % (n/n)	43.5 (10/23)	16.7 (4/24)
Maturation rate per follicle retrieved; % (n/n)	14.1 (10/71)	5.7 (4/70)
Diameters of MII oocytes (μm)	119 ± 3.2	120 ± 2.1

Proportions of ‘fast-growth’ follicles were evaluated at Week 3 of culture. Survival data were reported at Week 4 of culture. Rates were compared between follicles cultured *in vitro* with (IVG_NT) or without (IVG_C) supplementation of neurotrophic factor 4, with each follicle as an experimental unit. MII, meiosis II.

*  *P* < 0.05.

### DDX4 detection in human oocytes derived from IVG follicles

Oocytes harvested from IVG follicles exhibited a germinal vesicle nucleus (GV) and zona pellucida (ZP), surrounded by cumulus cells (CC), mirroring the characteristics of oocytes developed *in vivo*. As a widely accepted and evolutionarily conserved oocyte marker, DDX4 staining delineates its cytoplasmic localization in oocytes rather than CCs. DAPI staining depicts chromatin in the nucleus of oocyte and cumulus cells (Fig. 2C).

### Hormone production of human follicles during IVG

Estradiol-17 β, AMH, and progesterone were all detectable in the culture media from the first week of cultivation. From Week 3, estradiol and progesterone production were significantly increased in the NT4 group (Fig. 2F and G). However, throughout the *in vitro* culture, no significant difference was observed in the AMH production between the two groups (Fig. 2E).

### IVM of human IVG follicles

There were 24 follicles in the control group and 23 follicles in the NT4 group which underwent IVM. With IVM, metaphase II oocytes exhibiting normal morphology were successfully harvested, comprising the first polar body, cumulus cells, and zona pellucida with normal sizes and positions under optical microscopy (Fig. 1I). One of the metaphase II oocytes from the control group displayed vacuoles in the cytoplasm (Fig. 1J). None of the metaphase II oocytes in either group displayed an enlarged first polar body. The maturation rates per live follicle and per retrieved follicle in the control group were 16.7% (4/24) and 5.7% (4/70), producing MII oocytes with sizes of 120 ± 2.1 μm, while rates in the NT4 group were increased to 43.5% (10/23, *P* < 0.05 when compared with the control group) and 14.1% (10/71), producing MII oocytes with diameters of 119 ± 3.2 μm (Table 2).

### NT4 supplementation supports morphologically normal blastocyst formation

**Figure 3.**
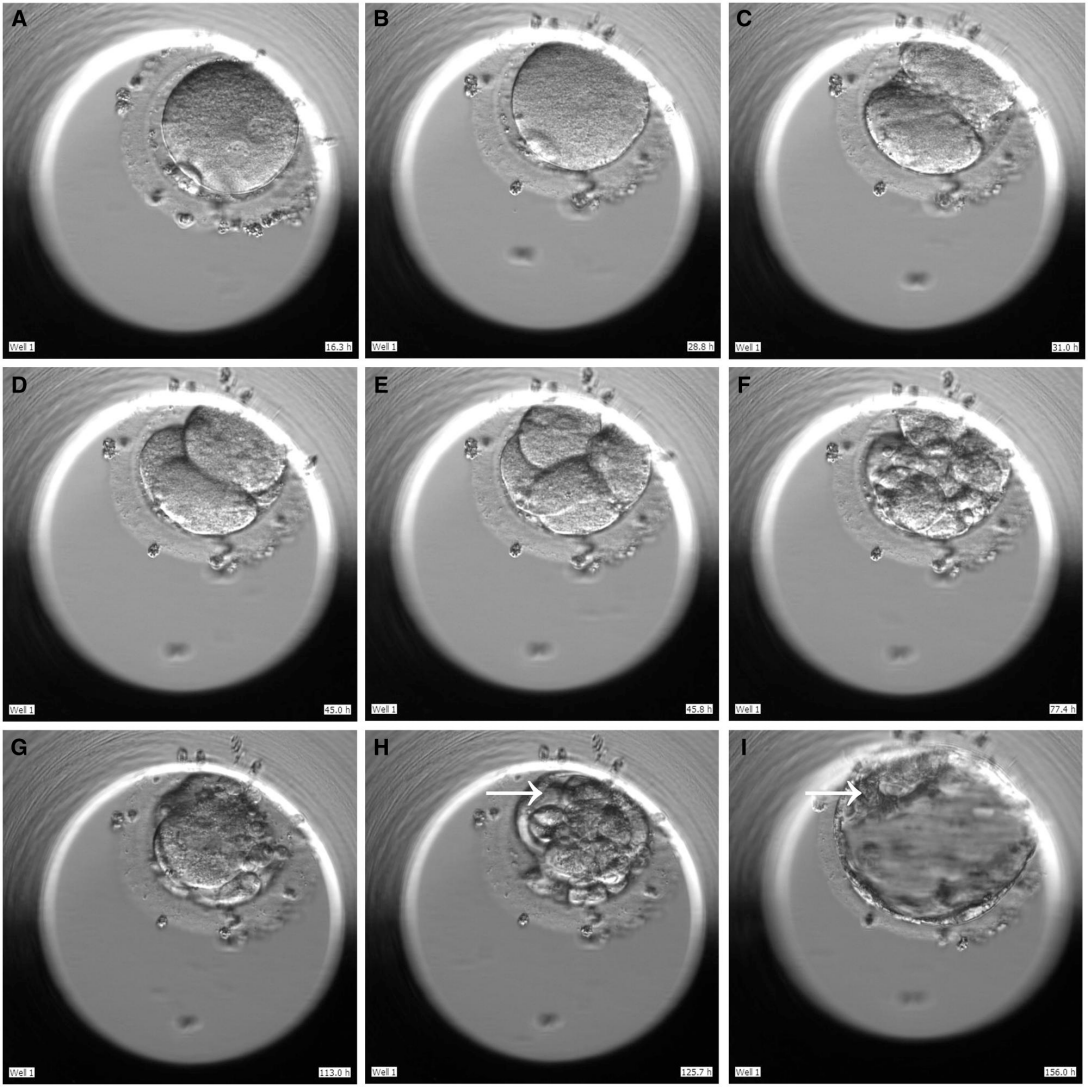
**Embryo development after fertilization of an oocyte harvested from human follicle cultured *in vitro* with neurotrophic factor 4 (NT4).** (**A**) Two pronuclei. (**B**) Pronuclei fade. (**C**) Two-cell embryo. (**D**) Three-cell embryo. (**E**) Four-cell embryo. (**F**) Eight-cell embryo. (**G**) Morula. (**H**) Appearance of blastocyst cavity (arrowhead). (**I**) Grade 4 blastocyst with loosely grouped inner cell mass (arrowhead) and a few cells in trophectoderm. The number in the lower right-hand corner of each image indicates hours since ICSI.

To test the reproductive potential of human IVG secondary follicles, two follicles (in the two groups respectively) from an 11-year-old patient (Patient No.1) were used for ICSI. Follicle A in the NT4 group grew from 179 to 821 μm in diameter within a 21-day IVG period (a ‘fast-growth’ follicle). During the same period, follicle B in the control group expanded from 201 to 896 μm (also a ‘fast-growth’ follicle). Both follicles were simultaneously transferred into the IVM medium. In the NT4 group (oocyte A), the first polar body was detected 28.5-h post-IVM, followed by ICSI utilizing donated sperm. The presence of 2PN was detected 16.3-h post-ICSI under optical microscopy (embryo A). The cellular behaviour of embryo A from the 2PN stage to blastocyst formation was dynamically captured by time-lapse imaging (Fig. 3 and Supplementary Video S1), which was graded as ‘8 cell with grade 2’ and ‘4BC’ on Day 3 and Day 5, respectively. The number on the lower right corner of each image indicates the hours elapsed post-ICSI. The morphokinetic variables are summarized in Supplementary Table S1.

**Figure 4.**
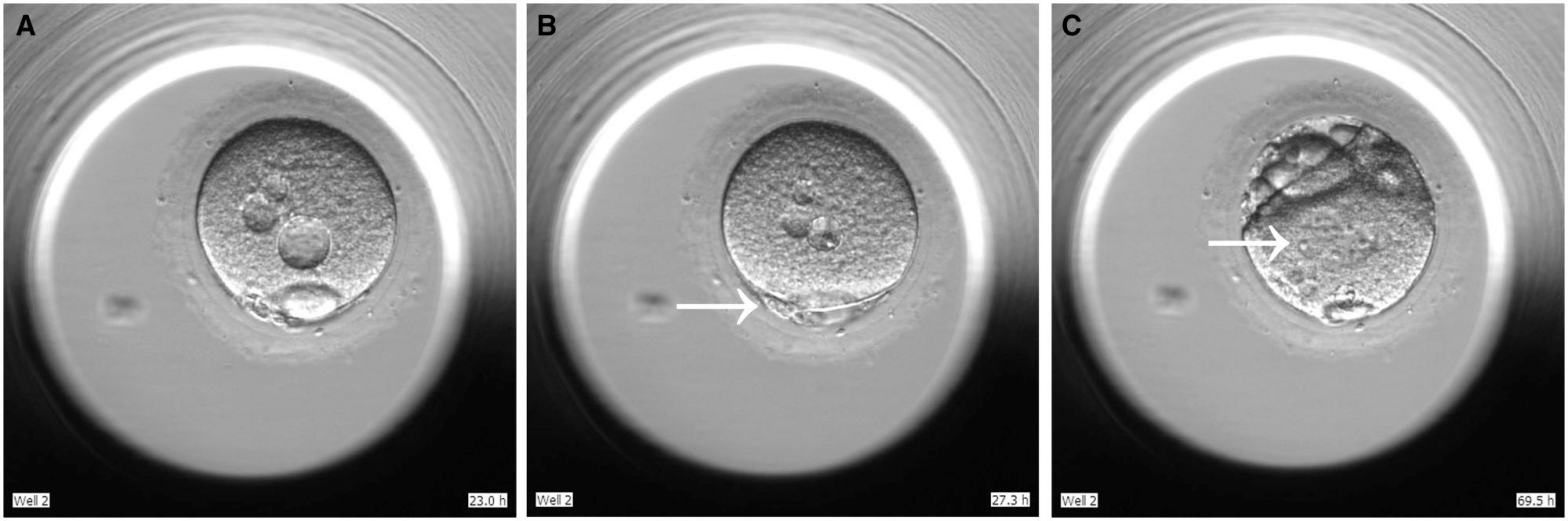
**Abnormal fertilization of an oocyte harvested from *in vitro* cultured human follicles in the control group.** (**A**) Fertilization of metaphase II oocytes with vacuoles in the cytoplasm was performed with ICSI. (**B**) The second polar body (arrowhead) was extruded. (**C**) Multiple pronuclei (arrowhead) were observed in the cytoplasm.

Cytoplasmic vacuoles were observed in the oocyte (oocyte B) from the control group. Subsequent to IVM, the first polar body was extruded after 44.8 h. ICSI was then performed with donated sperm from the same sample as used for oocyte A. Embryo B was transferred to the Embryoscope system for time-lapse imaging (Supplementary Video S2). (Note: Since embryo A and B were cultured in separated wells on the same Embryoscope system plate, and the ICSI time for embryo A had already been inputted for this plate, the time annotation in the lower right-hand corner of the images for embryo B images could not be revised and still indicates hours since oocyte A underwent ICSI, instead of oocyte B.) Figure 4A as well as the first image showing an embryo in Supplementary Video S2 could be regarded as the beginning of ICSI of embryo B. The second polar body was extruded but multiple pronuclei were observed (Fig. 4B and C).

## Discussion

Applying a matrix-free and long-term culture system, we constructed a human follicle IVG system enabling the development of mature oocytes, from secondary follicles isolated from fresh medulla tissue. Secondary follicles survived for up to six weeks with increasing amounts of steroid hormones, progressing to the antral stage and yielding MII oocytes with typical morphology. Despite similar initial diameters and survival rates, NT4 enhanced follicle growth with increased diameters and coordinated steroid hormone production. The proportion of ‘fast-growth’ follicles and the efficiency of MII oocyte production per live follicle were also significantly increased upon NT4 supplementation, resulting in 43% of the live follicles emitting a polar body. With this optimized IVG system, we describe here the successful development of a blastocyst with transfer potential derived from a prepubertal patient. Together, these results confirm the possibility of harvesting mature and fertilizable oocytes from human secondary follicle IVG.

Although healthy offspring have been produced following the culture of mouse secondary follicles, the advances have proved to be far less effective to replicate in humans with larger follicles and extended culture duration. To date, these challenges remain unresolved with only three culture systems reported capable of producing MII oocytes from human follicles, either from primordial stage (McLaughlin *et al.*, 2018; Xu *et al.*, 2021) or secondary stage (Xiao *et al.*, 2015) follicles. The final goal of a follicle IVG system is to generate oocytes capable of maturation and fertilization. However, the maturation efficiency of the human follicle IVG system (10.3–21% per alive follicles) remains unsatisfactory and a fertilizable oocyte remains unachieved.

The duration of growth and the span of follicle stages supported *in vitro* are important factors when optimizing the IVG system. Both the accelerated (McLaughlin *et al.*, 2018) (approximately 10 days) and long-term culture system (Xiao *et al.*, 2015; Xu *et al.*, 2021) (about 4–6 weeks) have successfully achieved mature oocytes, while the former resulted in enlarged polar bodies and smaller antral follicles (248 ± 4.5 μm) compared to the latter (600–700 μm). Notably, human folliculogenesis spans approximately 180 days *in vivo*, storing about 330 pg RNA in oocytes to provide vital materials for fertilization and embryogenesis (Paulino *et al.*, 2022). The IVG system needs to support follicles to accumulate enough of the necessary materials. In this study, the secondary follicles were isolated from fresh ovarian tissue, with a larger diameter (206 ± 61.3 and 184 ± 93.4 μm) than secondary follicles isolated from cultured ovarian tissue (∼120–223.2 μm) (McLaughlin *et al.*, 2018; Xu *et al.*, 2021) or *in vivo* developed ovarian tissue (165.8 ± 32.3 μm) (Xiao *et al.*, 2015), with the above study producing MII oocytes. Ultimately, follicles in this study survived up to 6 weeks, growing to antral sizes of almost 1 mm in diameter, which may be related to the improved oocyte maturation rates and capability to maintain development up to the blastocyst stage. In all, a long-term culture system supporting an IVG follicle to the stage of maximum maturity may be beneficial for subsequent oocyte maturation and embryo development.

To keep the follicles alive during a long-term IVG, the integrity of the basement membrane and 3-dimensional spherical structure needs to be maintained throughout the follicle culture before IVM. When isolating follicles for IVG, either with enzymatic digestion or mechanical separation, the integrity of the basement membrane should be protected. Until now, the evaluation of membrane integrity has mainly relied on the existence of a regular and complete shadow surrounding the follicle under the microscope. More accurate and non-invasive testing methods are needed. To maintain the architecture and the gap junction between the oocyte and granulosa cells, 3-dimensional culture systems were promoted (Xu *et al.*, 2009). Besides the use of encapsulation (Xiao *et al.*, 2015), matrix-free systems have shown effectiveness in maintaining the architecture and yielding mature oocytes (McLaughlin *et al.*, 2018; Xu *et al.*, 2021). In this study, the round bottom of the microplate, with its continuous annular platform, serves as a supportive structure for growing follicles of varying diameters. Besides, the ultra-low attachment surface pre-coated with specific material prevents the attachment of somatic cells, thus preserving the integrity of the basement membrane. Matrix-free systems also allow efficient diffusion of macromolecules and reduces operating time outside the incubator compared with the encapsulation system (Xu *et al.*, 2018a). While 3-dimensional imaging is unavailable in this study, the linear increase in follicle diameter and an exponential increase in steroid hormones production suggested an exponential proliferation of theca cells and granulosa cells within a 3-dimensional spherical structure.

During the *in vitro* development of the follicle before IVM, the secretion of estradiol-17 β and progesterone has been regarded as valid factors critical for antral follicle maturation (Skory *et al.*, 2015; Ting *et al.*, 2015; Baba *et al.*, 2017; Telfer *et al.*, 2023). AMH is also an excellent marker for the presence of growing follicles and the development of early antral follicle (Xu *et al.*, 2016, 2018a,b). When the follicles grew to the antral stage on Weeks 2–3 and transformed to being gonadotropin-dependent in this study, we observed an accelerated synthesis of steroid hormones. Along with the increased diameters in the NT4 group, the production of steroid hormones was also significantly superior compared to the control group. Though secreted by granulosa cells, the AMH displayed no difference between groups. The possible reasons are as follows: AMH production *in vitro* by early pre-antral follicles correlated positively with the growth rate, which reached a peak at the early antral follicle stage and declined at the large antral follicle stage. One media sample was collected weekly from each patient in this study, which means one media sample may incorporate production from both early antral follicles and large antral follicles, thereby hindering separate analysis of the effect of NT4 at different follicle stages. Besides, during the gonadotropin-dependent phase, the inhibitory effect of AMH on aromatase induction will gradually subside and consequent estradiol production in turn promotes the extinction of AMH in large antral follicle (Dewailly *et al.*, 2016). The adjustment between AMH, FSH, and androgens may prevent the overproduction of AMH in follicles of the NT4 group.

The ultimate goal of an IVG system is to produce mature oocytes that are competent for fertilization, while the appropriate timing and method of IVM after IVG need further exploration. The timing of IVM needs to be determined in combination with final follicle diameter, dynamic hormone secretion levels, and the follicle growth rate. Previous research has successfully obtained morphologically normal MII oocytes from human IVG follicles with terminal diameters of about 600∼1000 μm (Xiao *et al.*, 2015; Xu *et al.*, 2021). In this study, both groups reached average terminal diameters above 960 μm. On this basis, a higher growth rate of follicle correlated with an increased maturation rate of oocyte. The future progress in hormone detection for microsamples may help determine the optimal IVM timing for follicle IVG. For the method of IVM, previous studies cultured COCs retrieved from follicles instead of a whole follicle to achieve mature oocytes (Xiao *et al.*, 2015; McLaughlin *et al.*, 2018; Xu *et al.*, 2021). To mimic the *in vivo* situation, IVM was conducted with a whole follicle in this study and the effectiveness of attaining mature oocytes was confirmed, while it is not conducive to the morphological evaluation of cumulus expansion and mucification after IVM. Both the SAGE IVM medium (containing FSH and LH) (McLaughlin *et al.*, 2018; Xu *et al.*, 2021) and αMEM (with fetal bovine serum, hCG, EGF, and rFSH) (Xiao *et al.*, 2015) have been applied for IVM for various durations (24–48 h). Given that our previous studies (Zeng *et al.*, 2013; Gilchrist *et al.*, 2016) had shown the effect of cAMP modulation in improving IVM oocyte developmental competence, the pre-IVM medium with forskolin (cAMP modulation) was applied 4 h prior to IVM medium, resulting in a maturation rate per live follicle of 13/23 in the NT4 group.

After IVM, we further tested the embryogenesis potential of IVG follicles. Previously, the efficiency of human follicle IVG was defined by morphology, as well as molecular and metabolic factors. Fertility potential remained unknown. In this study, the potential of IVG to yield human oocytes competent to progress to blastocyst stage was confirmed. Though assessed as a ‘slow embryo’ according to the Istanbul consensus (Alpha Scientists in Reproductive Medicine and ESHRE Special Interest Group of Embryology, 2011), the embryo from the NT4 group went through the extended embryo culture which is regarded as an effective strategy to test implantation potential (McLernon *et al.*, 2016). Furthermore, the blastocyst was finally graded as 4BC, which means that it had transfer potential.

Among the above IVG processes, we had observed the promoting effect of NT4 as follows: despite similar initial diameters, terminal diameters, and survival rate, the IVG system supplied with NT4 showed a higher percentage of fast growth follicles, superior steroid hormone production, and an improved IVM rate compared with the control group, yielding a blastocyst with transfer potential. Folliculogenesis *in vivo* requires tight cooperation of autocrine, paracrine, and endocrine factors. AMH (Xu *et al.*, 2021) and activin (Telfer *et al.*, 2008) had been reported to improve follicle IVG from secondary stage to antral stage. However, the gain of mature oocytes remained unsatisfying. Previously (Guo *et al.*, 2021), we found that mice oocytes from IVG follicles are deficient in neuroendocrine regulation, and the supplementation of NT4 with a concentration of 100 ng/ml was shown to be superior in improving follicle diameters and oocyte maturation in secondary mice follicle IVG. We further substantiated the improvement in human secondary follicle IVG in the prescence of NT4 in this study. Consistent increases in follicle diameter and estradiol synthesis on the same observation day suggest that NT4 stimulates the proliferation of granulosa cells. Kerr *et al.* (2009) reported that NT4 enhanced *Fshr* gene expression and the response to FSH in murine ovaries. Additionally, both NT4 and its receptor have been detected in human oocytes and granulosa cells (Seifer *et al.*, 2006). These results lead to a hypothesis that NT4 may promote cell proliferation by upregulating FSH receptor expression or FSH reactivity in granulosa cells, thus enhancing IVG growth rate and maturation potential, which needs further validation.

Although pre-pubertal patients account for a large proportion of people with indications for fertility preservation, no MII oocyte harvested from IVG from a pre-pubertal patient has been clearly reported previously, potentially due to the physiological characteristic of follicles. Yin *et al.* (2016) reported that three of five secondary follicles obtained from fresh medullary tissue of a pre-pubertal patient developed to small antral follicles *in vitro*, while progressing to degeneration after a 28-day culture. Xiao *et al.* (2015) indicated that five of 19 pre-pubertal patients achieved secondary follicles for IVG. The cyclic recruitment (antral follicle to ovulation) is established in post-adolescent ovaries (Wallace and Kelsey, 2010). About two-thirds of the follicles in pre-pubertal mouse ovaries are degraded through follicular atresia, mediated by autophagy and apoptosis (Tingen *et al.*, 2009; Hułas-Stasiak and Gawron, 2011; Song *et al.*, 2015). Premenarchal patients exhibit higher rates of atretic follicles and decreased mature rates of IVM than postmenarchal women (Anderson *et al.*, 2014; Karavani *et al.*, 2019; Cacciottola *et al.*, 2023). The possibility of achieving mature and fertilizable oocytes through IVG of follicles from pre-pubertal patients has long been questioned. Though with a limited sample size, the results of this study confirm this possibility.

The population included in this study was all beta-thalassemia patients. Whether this culture system is effective for other patients remains unknown. Besides, follicles cultured *in vitro* were still smaller than that *in vivo*, with an unsatisfactory IVM rate. Constructing promising IVG for post- and pre-pubertal patients remains a major clinical challenge. Since the selected dose of NT4 was based on dose finding in mice, the optimal dose to use in a human IVG system needs further confirmation. Oocytes and embryos obtained from this study have not been examined for ploidy status and epigenetic signatures which are indispensable before therapeutic application.

We have shown that follicular development and the oocyte maturation potential of human secondary follicles IVG can be promoted by NT4. With the optimized system, the possibility of harvesting mature and fertilizable oocytes from IVG human secondary follicle was confirmed. Fresh medulla tissue obtained during tissue preparation for OTC, otherwise discarded, may serve as an additional precious source of fertilizable oocytes for fertility preservation, even for pre-pubertal girls, without the threat of tumour reintroduction. After further characterization and optimization of the system, it might also provide a powerful future research tool to investigate the mechanisms of human follicular development and to test for reproductive toxicity.

## Supplementary Material

hoae005_Supplementary_Data

## Data Availability

The data underlying this article are available in the article and the online supplementary material.
