# Supplementary material for: Neurotrophin-4 promotes in vitro development and maturation of human secondary follicles yielding metaphase II oocytes and successful blastocyst formation
Source: Hum Reprod Open. 2024 Jan 30;2024(1):hoae005. doi: 10.1093/hropen/hoae005 (PMC10873269; doi:10.1093/hropen/hoae005)
Supplement: hoae005_Supplementary_Data [file hoae005_supplementary_data.zip › Titles_and_legends_for_Supplementary_Videos_S1_and_S2_EO.docx]

**Supplementary Video S1. Embryonic formation from an oocyte harvested from a human follicle cultured *in vitro* with NT4.** The cellular behaviour of the embryo from the 2PN stage to blastocyst formation was recorded by time-lapse microscopy. (Note: The record includes two parts, as the time track of the embryo was interrupted for 0.9 hours due to the short power failure of the device.) The number in the lower right-hand corner of each image indicates hours since ICSI. ICSI, intracytoplasmic sperm injection.

**Supplementary Video S2. Abnormal fertilization of an oocyte harvested from an *in vitro* cultured human follicle in the control group.** The first image shown is the beginning of ICSI of the oocyte. ICSI, intracytoplasmic sperm injection.
