# Supplementary material for: Neurotrophin-4 promotes in vitro development and maturation of human secondary follicles yielding metaphase II oocytes and successful blastocyst formation
Source: Hum Reprod Open. 2024 Jan 30;2024(1):hoae005. doi: 10.1093/hropen/hoae005 (PMC10873269; doi:10.1093/hropen/hoae005)
Supplement: hoae005_Supplementary_Data [file hoae005_supplementary_data.zip › HRO-23-0161-R2-SuppTable1.docx]

**Supplementary Table S1. The** **morphokinetic variables of embryo A**

| Morphokinetic variables | Hours |
| --- | --- |
| t0 (time of ICSI) | |
| tPNa (appearance of individual pronuclei) | 16.3 |
| tPNf (pronuclei disappearance) | 28.8 |
| t2 (two discrete cells) | 31.0 |
| t3 (three discrete cells) | 45.0 |
| t4 (four discrete cells) | 45.8 |
| t8 (eight discrete cells) | 77.4 |
| tM (end of compaction process) | 113.0 |
| tSB (initiation of blastulation) | 125.7 |
